# On the Inclusion of Short-distance Bystander Effects into a Logistic Tumor Control Probability Model

**DOI:** 10.7759/cureus.2012

**Published:** 2018-01-01

**Authors:** David G Tempel, N. Patrik Brodin, Wolfgang A Tomé

**Affiliations:** 1 Department of Radiation Oncology, Montefiore Medical Center/Albert Einstein College of Medicine

**Keywords:** tcp, ntcp, bystander effect, selective boosting, radiation response modeling, statistical mechanics

## Abstract

Currently, interactions between voxels are neglected in the tumor control probability (TCP) models used in biologically-driven intensity-modulated radiotherapy treatment planning. However, experimental data suggests that this may not always be justified when bystander effects are important. We propose a model inspired by the Ising model, a short-range interaction model, to investigate if and when it is important to include voxel to voxel interactions in biologically-driven treatment planning. This Ising-like model for TCP is derived by first showing that the logistic model of tumor control is mathematically equivalent to a non-interacting Ising model. Using this correspondence, the parameters of the logistic model are mapped to the parameters of an Ising-like model and bystander interactions are introduced as a short-range interaction as is the case for the Ising model. As an example, we apply the model to study the effect of bystander interactions in the case of radiation therapy for prostate cancer. The model shows that it is adequate to neglect bystander interactions for dose distributions that completely cover the treatment target and yield TCP estimates that lie in the shoulder of the dose response curve. However, for dose distributions that yield TCP estimates that lie on the steep part of the dose response curve or for inhomogeneous dose distributions having significant hot and/or cold regions, bystander effects may be important. Furthermore, the proposed model highlights a previously unexplored and potentially fruitful connection between the fields of statistical mechanics and tumor control probability/normal tissue complication probability modeling.

## Introduction

Within the last decade there has been renewed interest in intensity-modulated radiotherapy (IMRT) strategies based on biological optimization [1-4]. Biologically-driven IMRT is particularly promising for targeting inhomogeneous dose distributions to tumors with multiple risk regions, [5-8] in which the dose within the tumor is redistributed from low risk regions to high risk regions allowing one to increase the therapeutic ratio as compared to traditional IMRT techniques that tend to deliver a homogenous dose distribution to the entire tumor​​​​​​​. Given their potential, biologically-driven optimization routines are now available in several treatment planning software packages for research purposes, with the goal that they will become accurate enough for use in the clinic [9-10].

Unlike traditional IMRT based on physical constraints, the central quantities in biologically-driven IMRT are probabilities, namely the tumor control probability (TCP) and normal tissue complication probability (NTCP). Therefore, an accurate biologically-driven IMRT strategy requires models of TCP and NTCP, which can correctly capture the statistics of the underlying radiobiology. Several models such as the logistic model [11-13] and the Poisson linear-quadratic model [14] currently exist, but these models make the following three assumptions [15]: 1) each tumor consists of a number of noninteracting clonogens, 2) clonogen killings are uncorrelated events, 3) a tumor is controlled if all its clonogens are inactivated (sterilized). The third assumption, known as the “clonogen hypothesis” is intuitive and generally accepted. However, experiments have shown that the first and second assumptions are not always justified. Indeed, it has been observed that irradiated tumor regions can radio-sensitize other regions at distant sites [16-20]. Such interactions between clonogens are called “bystander interactions” and have been a subject of much study in recent years [21-24]. In order to investigate whether or not bystander interactions are important in biologically-driven IMRT, it is desirable to construct TCP and NTCP models, which are capable of going beyond the non-interacting voxel assumption.

In searching for such interacting models, it is natural to make a connection with statistical mechanics, which addresses problems in theoretical physics that superficially appear very different from radiobiology, but mathematically are quite similar. In statistical mechanics, one deduces the macroscopic phases of matter, given a probabilistic model of the interacting microscopic components. For instance, in the statistical mechanics of magnetism, one is interested in whether a material will macroscopically behave as a ferromagnet or a paramagnet at a given temperature and magnetic field. To answer this question, one writes down a microscopic two-state model describing the interactions of the atoms with an external magnetic field and with each other, which is known as the Ising model. One then computes the probabilities that the atoms will have their magnetic moments all aligned (ferromagnetic state). At its most basic level, biologically-driven IMRT is concerned with a similar problem, the desirable state being that all clonogens are sterilized. Given a set of biological parameters, which characterize the interaction of tumor clonogens with a radiation dose and possibly bystander interactions with each other, one is interested in the probability that all clonogens are sterilized, i.e. $TCP(D)=1.0$.

In the present manuscript, we explore and exploit the connection between biologically-driven IMRT and statistical mechanics by first showing that the widely-used logistic TCP model is mathematically equivalent to a non-interacting Ising model. Having established this connection between the non-interacting models, we proceed to construct an interacting “Ising-like” TCP model, which builds on the non-interacting logistic model, into which bystander interactions can be explicitly incorporated as a short-range interaction. The interactions are modeled by assuming that radiation-damaged cells release a diffusible chemical mediator that radio-sensitizes adjacent cells, which is a widely accepted mechanism of the bystander effect [21, 23]. As a proof of concept, the model is used to study the effect of bystander interactions in a simplified example of prostate cancer treatment. Given the experimental uncertainty in the model parameters, we do not attempt to make quantitative predictions. However, we are able to draw important qualitative conclusions by studying the model in a variety of parameter regimes.

## Materials and methods

1. Background

In this section, we review the Ising model and the logistic model for TCP and NTCP evaluation. The reader already familiar with these topics may wish to skip ahead to section 2, where we draw the connection between these two models.

1.1 The Ising model

In its original formulation, the Ising model was used to describe magnetic materials [25]. Due to its mathematical generality, it has since been applied to a vast array of different systems in chemistry, physics, and biology. However, for concreteness we will focus the present discussion on the original application of the model, which describes the *collective behavior* of $N$ particles arranged on a lattice that each can assume one of *two* mutually exclusive states, interacting with one another through ferromagnetic forces as well as an external magnetic field. The magnetic field is characterized by a set of $N$ numbers, $\{B\} \equiv \{ B_1, B_2, ...B_N \}$, which specify its magnitude at each lattice point. Similarly, the state of the particles is described by a set of binary variables, $\{ \sigma \} \equiv \{ \sigma_1, \sigma_2, ...\sigma_N \}$, which specify the direction of the magnetic moment at each lattice point. If $\sigma_i = +1$, the ith particle has its magnetic moment aligned parallel (anti-parallel) to the magnetic field when $B_i >0$ ($B_i &lt;0$), while if $\sigma_i = -1$ it is parallel (anti-parallel) when $B_i &lt;0$ ($B_i >0$). A particle with $\sigma_i = +1$ is said to be “spin-up”, while one with $\sigma_i = -1$ is “spin-down.” There exist $2^N$ different configurations, $\{ \sigma \}$, and the energy of a given configuration is given by:

$E(\{ \sigma \}) = -\sum_{i = 1}^N \sum_{j = 1}^N J_{i,j} \sigma_i \sigma_j - \sum_{i = 1}^N B_i \sigma_i,$    (1)

where $J_{i,j}$ is a positive number, which specifies the strength of the interaction between particles at the ith and jth lattice sites. The first term in equation (1) is minimized when all particles have their magnetic moments aligned parallel to one another and thus describes a ferromagnetic interaction. The second term is minimized when all particles are aligned parallel to the external magnetic field. Therefore, at absolute-zero of temperature where the particles are in their lowest energy state, all particles are parallel to each other and the external magnetic field. At finite temperatures, particles have a non-zero probability to flip their orientation and one must rely on statistical mechanics to compute the probabilities of different configurations.

According to statistical mechanics, the probability $P(\{ \sigma \})$ of finding the lattice in a given configuration at temperature $T$ is given by the Boltzmann distribution (cf. Ref. [26]),

$P(\{ \sigma \}) =\frac{1}{Z} e^{-\frac{E(\{ \sigma \})}{k_B T}}.$    (2)

Here, $k_B=1.38 \times 10^{-23}$ is Boltzmann's constant measured in units of joules per degree Kelvin and

$Z = \sum_{\{ \sigma \}}e^{-\frac{E(\{ \sigma \})}{k_B T}},$    (3)

is the partition function. In equation (3) we have used the notation $\sum_{\{ \sigma \}} \equiv \sum_{\sigma_1} \sum_{\sigma_2}... \sum_{\sigma_N}$ to denote the sum over all lattice configurations. According to equation (2), large values of the parameter sets $\{ J_{i,j} \}$ and $\{ B_i \}$ with small $T$ favor an ordered (ferromagnetic) state, with all particles aligned in the same direction. On the other hand, small $\{ J_{i,j} \}$ and $\{ B_i \}$ with large $T$ favor a disordered (paramagnetic) state. In between these two extremes, a phase transition from the ordered to the disordered state occurs.

The partition function defined in equation (3) plays a central role in statistical mechanics, providing a link between the microscopic state of particles and macroscopically measured quantities such as the entropy, average energy, and pressure. This is accomplished by defining the Helmholtz free energy in terms of the logarithm of the partition function as:

$F \equiv -k_B T \ln(Z).$    (4)

The thermodynamic quantities are then given by derivatives of the free energy. Accordingly, the entropy, internal energy, and pressure are found as follows (cf. Ref. [26]):

$S = -\left.\frac{\partial F}{\partial T}\right|_{V},$    (5)

$U = -T^2 \left.\frac{\partial (F / T)}{\partial T}\right|_{V},$    (6)

and

$P = -\left. \frac{\partial F}{\partial V}\right|_{T},$   (7)

respectively, where $V$ denotes the volume of the system.

The different phases of the Ising model are understood by studying its correlation functions, which are calculated from derivatives of the free energy with respect to the magnetic field. The one-point correlation function or local magentization is defined for the ith lattice site as,

$C_{i}^{(1)} \equiv \sum_{\{ \sigma \}} P(\{ \sigma \}) \sigma_i = - \frac{\partial F}{ \partial B_{i}}.$   (8)

Similarly, one can define the two-point correlation function between the ith and jth sites as,

$C_{ij}^{(2)} \equiv \sum_{\{ \sigma \}} P(\{ \sigma \}) \sigma_i \sigma_j = \frac{\partial^2 F}{ \partial B_{i} \partial B_{j}}$   (9)

and the N-point correlation function for the entire lattice,

$C^{(N)} \equiv \sum_{\{ \sigma \}} P(\{ \sigma \}) \left[ \prod_{i=1}^{N} \sigma_i \right] = (-1)^{N} \frac{\partial^{N} F}{ \partial B_{1} \partial B_{2} ... \partial B_{N}}.$    (10)

The “order parameter” is defined by taking the average of the one-point correlation functions:

$C^{(1)}_{avg} \equiv \frac{1}{N} \sum_{i=1}^{N} C_{i}^{(1)}.$    (11)

$C^{(1)}_{avg} = \pm 1$ in a perfectly ordered phase (ferromagnetic) and $C^{(1)}_{avg} = 0$ in a completely disordered phase (paramagnetic). The decay of the two-point correlation with the inter-particle distance yields information about the correlation length, which characterizes the size of magnetically ordered domains. The N-point correlation function is generally not studied in magnetism, but will be relevant in section 2.1, where it will be shown to be equivalent to TCP.

1.2 The logistic model of tumor control

The logistic model is a widely used model to describe tumor and normal tissue dose response [11-13]. It also serves as a convenient model for making connections with the Ising model. We consider a tumor consisting of $N$ voxels, and $R$ different risk regions. Assuming that the dose response of the different risk regions are not correlated, the tumor control probability (TCP) for the entire tumor is written as a product of the TCPs for each risk region according to,

$TCP = \prod_{j=1}^{R} TCP_{j}^{v_j},$   (12)

where $v_j =\frac{V_j}{V} = \frac{N_j}{N}$ is the fraction of tumor voxels (or volume fraction) in the jth region, consisting of $N_j$ voxels. Furthermore, assuming that the dose response of the individual voxels is uncorrelated, the TCP of the jth risk region is a product of the TCPs of each voxel:

$TCP_j = {\left[\prod_{i=1}^{N_j} TCP_{i,j}\right]}^{\frac{1}{N_{j}}}.$    (13)

In the logistic model, the TCP of each voxel is modeled as,

$TCP_{i,j} = \frac{1}{1 + e^{-\left[ \frac{4 \gamma_{50}^j}{D_{50}^j}\right] (D_{i}^j - D_{50}^j) }} = \frac{e^{-\left[ \frac{4 \gamma_{50}^{j}}{D_{50}^{j} }\right] (D_{50}^{j} - D_{i}^{j})}}{1 + e^{-\left[ \frac{4 \gamma_{50}^{j}}{D_{50}^{j}}\right] (D_{50}^{j} - D_{i}^{j}) }},$    (14)

where $D_{i}^{j}$ is the dose received by the ith voxel in the jth risk region, $D_{50}^{j}$ is the dose at which 50 percent of tumor clonogens are sterilized and $\gamma_{50}^{j}$ is the slope of the dose response curve at $D_{i}^{j} = D_{50}^{j}$.

In writing the product form in equation (13), it is essential to assume that the TCP of each voxel is independent of all other voxels. In section 2.3 we will see that when bystander effects are included, this is no longer valid because the TCP of an individual voxel will be correlated with the TCP of all other voxels in the tumor.

2. Connection between the Ising model and the logistic model of tumor control

Having separately reviewed the Ising model and the logistic model of tumor control we will now connect these two models. We begin in section 2.1 by showing that the logistic TCP model can be rigorously formulated as a non-interacting Ising model, with an appropriate mapping between parameters. While not explicitly shown, by analogy the same is true for a logistic NTCP model. In section 2.2, we discuss how this mapping connects concepts from statistical mechanics such as temperature, energy, and phase transitions to TCP/NTCP modeling. Finally, in section 2.3 we build on this connection to include bystander interactions in the logistic model as an Ising-like interaction. For simplicity, all equations will be derived assuming a single risk region ($R = 1)$ in equation (12), but the generalization to multiple risk regions is straightforward.

2.1 Mapping between the logistic model and Ising model for non-interacting voxels 

According to the clonogen hypothesis, a voxel is sterilized if and only if all clonogens in the voxel are sterilized. This hypothesis implies that each tumor voxel can be regarded as a probabilistic system with two possible states. For the ith voxel, these two states are: 1) The voxel is sterilized with probability $TCP_{i}$ or 2) the voxel is not sterilized with probability $1-TCP_{i}$, where $TCP_{i}$ is given by equation (14) for a single risk region. To make the connection with statistical mechanics, we introduce a binary random variable $\sigma_i$, where $\sigma_i=1$ corresponds to the ith voxel being completely sterilized, while $\sigma_i=0$ corresponds to clonogens in the voxel remaining. In terms of this binary variable, the TCP of the ith voxel is simply the statistical average of $\sigma_i$:

$TCP_{i} = \sum_{\sigma_{i} = 0,1 }P_{i} (\sigma_{i})\sigma_{i},$    (15)

with the probability distribution,

$P_{i} (\sigma_i) = \left[ \frac{1}{1 + e^{-\left[ \frac{4 \gamma_{50}}{D_{50}}\right] (D_{50} - D_{i}) }} \right] e^{-\left[ \frac{4 \gamma_{50}}{D_{50}}\right] (D_{50} - D_{i}) \sigma_i}.$     (16)

One can explicitly verify that substituting equation (16) into equation (15) yields equation (14) for the TCP of the ith voxel. For non-interacting voxels, the probability distribution for all $N$ voxels is simply a product of the single voxel distributions according to:

$P(\{ \sigma \}) = \prod_{i=1}^{N} P_{i} (\sigma_i) = \frac{e^{-\left[ \frac{4 \gamma_{50}}{D_{50}}\right] \sum_{i=1}^N (D_{50} - D_{i}) \sigma_i}}{\prod_{i=1}^{N} \left[ 1 + e^{-\left[ \frac{4 \gamma_{50}}{D_{50}}\right] (D_{50} - D_{i}) }\right] } = \frac{e^{-\left[ \frac{4 \gamma_{50}}{D_{50}}\right] \sum_{i=1}^N (D_{50} - D_{i})  \sigma_i}}{\sum_{\{ \sigma \}} e^{-\left[ \frac{4 \gamma_{50}}{D_{50}}\right] \sum_{i=1}^N (D_{50} - D_{i})  \sigma_i} },$    (17)

where we use the notation, $\sum_{\{ \sigma \}} \equiv \sum_{\sigma_1 = 0,1 } \sum_{\sigma_2 = 0,1 }, ... \sum_{\sigma_N = 0,1 }$. In arriving at the final equality in equation (17), we have rewritten the denominator using the following manipulations:

$\prod_{i=1}^{N} \left[ 1 + e^{-\left[ \frac{4 \gamma_{50}}{D_{50}}\right] (D_{50} - D_{i})}\right] = \prod_{i=1}^{N} \left[ e^{-\left[ \frac{4 \gamma_{50}}{D_{50}}\right] (D_{50} - D_{i})(0)} + e^{-\left[ \frac{4 \gamma_{50}}{D_{50}}\right] (D_{50} - D_{i})(1) }\right] = \prod_{i=1}^{N} \left[ \sum_{\sigma_i = 0,1 } \ e^{-\left[ \frac{4 \gamma_{50}}{D_{50}}\right] (D_{50} - D_{i})\sigma_i } \right]$

$= \sum_{\sigma_1 = 0,1 } \ e^{-\left[ \frac{4 \gamma_{50}}{D_{50}}\right] (D_{50} - D_{1})\sigma_1} \sum_{\sigma_2 = 0,1 } \ e^{-\left[ \frac{4 \gamma_{50}}{D_{50}}\right] (D_{50} - D_{2})\sigma_2}... \sum_{\sigma_N = 0,1 } \ e^{-\left[ \frac{4 \gamma_{50}}{D_{50}}\right] (D_{50} - D_{N})\sigma_N}$

$= \sum_{\sigma_1 = 0,1 } \sum_{\sigma_2 = 0,1 }...\sum_{\sigma_N = 0,1 } e^{-\left[ \frac{4 \gamma_{50}}{D_{50}}\right] \sum_{i=1}^N (D_{50} - D_{i}) \sigma_{i}} = \sum_{\{ \sigma \}} e^{-\left[ \frac{4 \gamma_{50}}{D_{50}}\right] \sum_{i=1}^N (D_{50} - D_{i})  \sigma_{i}} .$      (18)

The TCP for all N voxels is obtained from the probability distribution according to,

$TCP= \left[ \sum_{\{ \sigma \}} P(\{ \sigma \})\left[ \prod_{i=1}^{N} \sigma_i \right] \right]^{\frac{1}{N}},$    (19)

To arrive at equation (19), we first use equations (12), (13), and (15) to write the TCP as,

$TCP = \left[ \prod_{i=1}^{N} TCP_{i} \right]^{\frac{1}{N}} = \left[ \prod_{i=1}^{N} \sum_{\sigma_i = 0,1 } P_{i} (\sigma_{i}) \sigma_{i} \right]^{\frac{1}{N}},$    (20)

and then use the following manipulations:

$\left[ \prod_{i=1}^N \sum_{\sigma_i = 0,1 } P_{i} (\sigma_i) \sigma_i \right]^{\frac{1}{N}} = \left[ \sum_{\sigma_1 = 0,1 } P_1 (\sigma_1) \sigma_1 \sum_{\sigma_2 = 0,1 } P_2 (\sigma_2) \sigma_2\hspace{1mm} ...\sum_{\sigma_N = 0,1 } P_N (\sigma_N) \sigma_N \right]^{\frac{1}{N}}$

$= \left[ \sum_{\sigma_1 = 0,1 } \sum_{\sigma_2 = 0,1 } ... \sum_{\sigma_N = 0,1 } P_1 (\sigma_1) P_2 (\sigma_2) \hspace{1mm} ... P_N (\sigma_N)\sigma_1 \sigma_2 ... \sigma_N \right]^{\frac{1}{N}}$

$= \left[ \sum_{\sigma_1 = 0,1 } \sum_{\sigma_2 = 0,1 } ... \sum_{\sigma_N = 0,1 } \left[ \prod_{i=1}^N P_i(\sigma_i) \right] \left[ \prod_{i=1}^N \sigma_i \right] \right]^{\frac{1}{N}} = \left[ \sum_{\{ \sigma \}} P(\{ \sigma \})\left[ \prod_{i=1}^N \sigma_i \right] \right]^{\frac{1}{N}}.$    (21)

Comparing equation (17) with equations (1) and (2), we see that the logistic model written in terms of the set of binary variables, $\{ \sigma \} \equiv \{ \sigma_1, \sigma_2, ...\sigma_N \}$, is in fact identical to the non-interacting Ising model, by introducing the following correspondences:

$B_i \equiv D_{i} - D_{50},$    (22)

$k_{B} T\equiv \frac{D_{50}}{4 \gamma_{50}},$    (23)

and a redefinition of the domain of the binary variables from $\sigma_i = \pm 1$ to $\sigma_i = 0,1$.

Comparison of equation (15) to (8) shows that the single-voxel TCP in the logistic model is equivalent to the one-point correlation function in the Ising model. Similarly, comparison of equation (19) to equation (10) shows that the full N-voxel TCP is equivalent to the N-point correlation function raised to the power $\frac{1}{N}$.

2.2 Analogy between TCP/NTCP modeling and statistical mechanics

The mapping between the logistic and non-interacting Ising models provides a formal connection between statistical mechanics and TCP/NTCP modeling. From a comparison of equation (17) with equations (1) and (2), together with the correspondences defined in equations (22) and (23), we see that for a given configuration of voxels, $\{ \sigma_{1}, \sigma_{2}, ...\sigma_{N} \}$, it is possible to define a “tumor energy” according to,

$E(\{ \sigma \}) \equiv \sum_{i=1}^N (D_{50} - D_{i}) \sigma_{i}.$    (24)

The quantity $(D_{50} - D_{i}) \sigma_{i}$ can be regarded as the energy of the ith voxel and we see from equation (22), that $D_{i} - D_{50}$ plays the role of the external magnetic field in the Ising model. In the non-interacting Ising model the lowest energy or “ground state” is a configuration, in which the ith spin is spin-down for $B_{i} &lt; 0$ and spin-up for $B_{i} > 0$. Similarly, for $D_{i} &lt; D_{50}$ the ground state corresponds to the ith voxel being unsterilized, while for $D_{i} > D_{50}$ the ith voxel is sterilized. Thus, if the dose distribution is such that $D_{i} > D_{50}$ for all voxels, the ground state corresponds to the configuration in which all voxels are sterilized.

In the Ising model at low temperatures, the ground state configuration dominates and all spins are aligned parallel to the magnetic field with near certainty. At higher temperatures, there is a non-zero probability for excited configurations to be realized, in which spins are oriented opposite to the magnetic field. From the mapping in equation (23), we see that a steep dose response (large $\gamma_{50}$) in the logistic model corresponds to a low ``tumor temperature" implying that the lowest energy configuration dominates. In this limit, a voxel is unsterilized with near certainty when $D_{i} &lt; D_{50}$ and it is sterilized with near certainty when $D_{i} > D_{50}$. Thus, for low tumor temperatures it is sufficient to have $D_{i} > D_{50}$ at all voxels to be confident that the tumor is controlled. In the limit $\gamma_{50} \rightarrow \infty$, there is a discontinuity in the dose response curve at the point $D_{i} = D_{50}$. In the language of statistical mechanics, this point corresponds to a zero temperature phase transition between two ordered phases (completely unsterilized and completely sterilized).

In contrast, a shallow dose response (small $\gamma_{50}$) corresponds to large tumor temperature, where many unsterilized voxels may still exist, even when $D_{i} > D_{50}$ is satisfied for all voxels. Thus, in the same way that temperature determines the degree of uncertainty in the state of a system in statistical mechanics, the quantity $\frac{1}{\gamma_{50}}$ determines the uncertainty that all tumor clonogens are successfully sterilized. In this case, there is no longer a sharp transition at $D_{i} = D_{50}$ for non-interacting voxels. However, as we shall see below when voxel-voxel interactions are included, a sharp phase transition from complete unsterilization, to complete sterilization occurs even for small $\gamma_{50}$. This is in some respects analogous to the disappearance of the paramagnetic phase that occurs in magnetic materials below the curie temperature [27-28].

By determining the steepness of the dose response curve, the tumor temperature T, in equation (23) determines the amount of dose in excess of $D_{50}$ required to be certain that all tumor clonogens are sterilized. It can therefore be used to provide an intuitive definition of “hot” and “cold” spots in the dose distribution [29]. We define a local temperature for the ith voxel as, $T_i \equiv \frac{D_{i} -D_{50}}{k_B}$. The TCP for this voxel can then be written in terms of the local and tumor temperatures as,

$TCP_{i} = \frac{1}{1+ e^{-\frac{T_i}{T}}}.$    (25)

A “hot” voxel is controlled with high probability ($TCP \approx 1$), which therefore satisfies $T_i \gg T$, while a “cold” voxel has $T_i$ close to or less than $T$. Clearly, this definition of hot and cold is not unique, as one can define a tumor temperature with respect to other measures, such as the average dose or effective uniform dose [30].

In analogy to equation (3) using the identifications given in equations (22) and (23), it is natural to define the denominator in equation (17) as the tumor partition function:

$Z \equiv \sum_{\{ \sigma \}} e^{-\left[ \frac{4 \gamma_{50}}{D_{50}} \right] \left[ \sum_{i=1}^{N}(D_{50} - D_{i}) \sigma_{i} \right]}.$    (26)

With this definition, it is clear that one can define a tumor free energy, entropy, pressure, internal energy, correlation functions, and order parameter in an entirely analogous way to equations (4) - (11).

2.3 Including bystander interactions

Having demonstrated the correspondence between the logistic TCP model and the non-interacting Ising model in section 2.1, we will now proceed to include the effect of bystander interactions. Because the two models are identical at the non-interacting level, we expand on this correspondence by suggesting the inclusion of bystander interactions by adding an “Ising-like” interaction term to equation (17). Thus, the probability distribution of N interacting voxels is given by:

$P (\{ \sigma \}) = \frac{1}{Z} e^{-\left[ \frac{4 \gamma_{50}}{D_{50}} \right] \left[ - \sum_{{i,j}}^{N} J_{ij} \sigma_i \sigma_j + \sum_{i=1}^{N}(D_{50} - D_{i}) \sigma_i \right]},$   (27)

where,

$Z = \sum_{\{ \sigma \}} e^{-\left[ \frac{4 \gamma_{50}}{D_{50}} \right] \left[ - \sum_{{i,j}}^{N} J_{ij} \sigma_i \sigma_j + \sum_{i=1}^{N}(D_{50} - D_{i}) \sigma_i \right]},$    (28)

is the interacting tumor partition function. The parameters $J_{ij}$ characterize the strength and range of the bystander interaction. Equation (27) is actually more than just an intuitive choice, it is one of the most general binary models that one can construct with two-voxel interactions, which reduces to the logistic TCP model in the non-interacting limit.

The single-voxel TCP is still given by equation (15) when interactions are included using the probability distribution in equation (27), provided one uses the marginal probability distribution,

$P_{i}(\sigma_i) = \sum_{\sigma_1 = 0,1 },... \sum_{\sigma_{i-1} = 0,1 } \sum_{\sigma_{i+1} = 0,1 }, ... \sum_{\sigma_N = 0,1 } P (\{ \sigma \}),$   (29)

constructed by summing the N-voxel probability distribution over all but the ith voxel. Equation (29) reduces to equation (16) for uncorrelated voxels. It follows that the N-voxel TCP is still given by equation (19) using the probability distribution in equation (27). However, because equation (27) is not a product of single-voxel distributions the relation, $TCP = \left[ \prod_{i=1}^N TCP_{i} \right]^{\frac{1}{N}}$ no longer holds, i.e. the probability that a given voxel is sterilized is now correlated with the state of the other voxels in the tumor.

In the Ising model, the microscopic physics of the magnetic interactions are encoded in the set of numbers $\{J_{ij} \}$. Similarly, in equation (27) the underlying biology of bystander interactions between voxels are encoded in the numbers $\{ J_{ij} \}$. In the present work, we choose to model the bystander interaction between voxels by the equation,

$J_{ij} =J_0 TCP_{i} e^{-\frac{|\vec{r}_i - \vec{r}_j|}{\lambda}},$    (30)

where $J_0$ and $\lambda$ are positive, real numbers, which respectively characterize the strength and range of the bystander interaction. $\vec{r}_i$ and $\vec{r}_j$ are the position vectors of the ith and jth voxels, respectively. In this model, $J_{ij}$ characterizes the radio-sensitization experienced by the jth voxel, due to its interaction with the ith voxel. Equation (30) is biologically motivated by the assumption that a voxel containing damaged cells releases diffusible factors, which tend to radio-sensitize cells in nearby voxels. The quantity of diffusible factor released by the ith voxel is proportional to the probability that it is sterilized ($TCP_{i}$). The degree of sensitization experienced by the jth voxel due to the ith voxel, decays exponentially with a characteristic distance $\lambda$. This dependence on distance was chosen because it is a simple (characterized by a single distance parameter $\lambda$) and generic short-range interaction. The interaction in equation (30) is clearly radio-sensitizing, because the probability that $\sigma_i = \sigma_j = 1$ (both voxels sterilized) in equation (27) is enhanced for larger values of $J_{ij}$. The parameters can be extrapolated from experimental data, or calculated from a microscopic model of diffusion. The data in Ref. [20] indicates that $\lambda \approx 1$ mm. To estimate $J_0$ from the experimental data, an independent estimate of $D_{50}$ and $\gamma_{50}$ would be needed.

Note that the bystander interaction introduced in equation (30) is inherently non-linear. Mathematically, this arises because the interaction $\{ J_{ij} \}$ depends on the probability distribution through $TCP_{i}$, but $TCP_{i}$ in turn depends on $\{ J_{ij} \}$ through equation (15) and equations (27), (28) and (29). Biologically, this is because the bystander interaction depends on the probability of cell-kill, but the probability of cell-kill in turn depends on the bystander interactions. Although in principle the non-linear problem can be solved, in practice one can linearize the interaction by replacing $TCP_{i}$ in equation (30) by the non-interacting TCP, i.e. its zeroth-order approximation, yielding

$J_{ij} \approx J_0 \left[ \frac{1}{1 + e^{-\left[ \frac{4 \gamma_{50}}{D_{50}}\right] (D_{i} - D_{50}) }} \right] e^{-\frac{|{\vec{r}}_{i} - {\vec{r}}_{j}|}{\lambda}}.$    (31)

The numerical implementation of the model is greatly simplified by regarding $J_{ij}$ to be an explicit function of the dose as in equation (31), rather than the TCP as in equation (30). This approximation is valid under the reasonable assumption that diffusible factors are primarily released in response to radiation-induced cell killing and not bystander-induced cell killing.

## Results

As a proof of principle, we study the bystander interaction model presented in section 2.3 using a simplified example of biologic-based IMRT treatment planning for prostate cancer. Given the current lack of definitive experimental evidence pertaining to the bystander model parameters, our goal is to study the model over a range of parameters to gain general qualitative insights. We begin with a brief overview of the numerical implementation of the model. Then we study the implications of bystander interactions when a nearly homogeneous dose distribution is delivered to a target.

Numerical implementation

**Figure 1:**
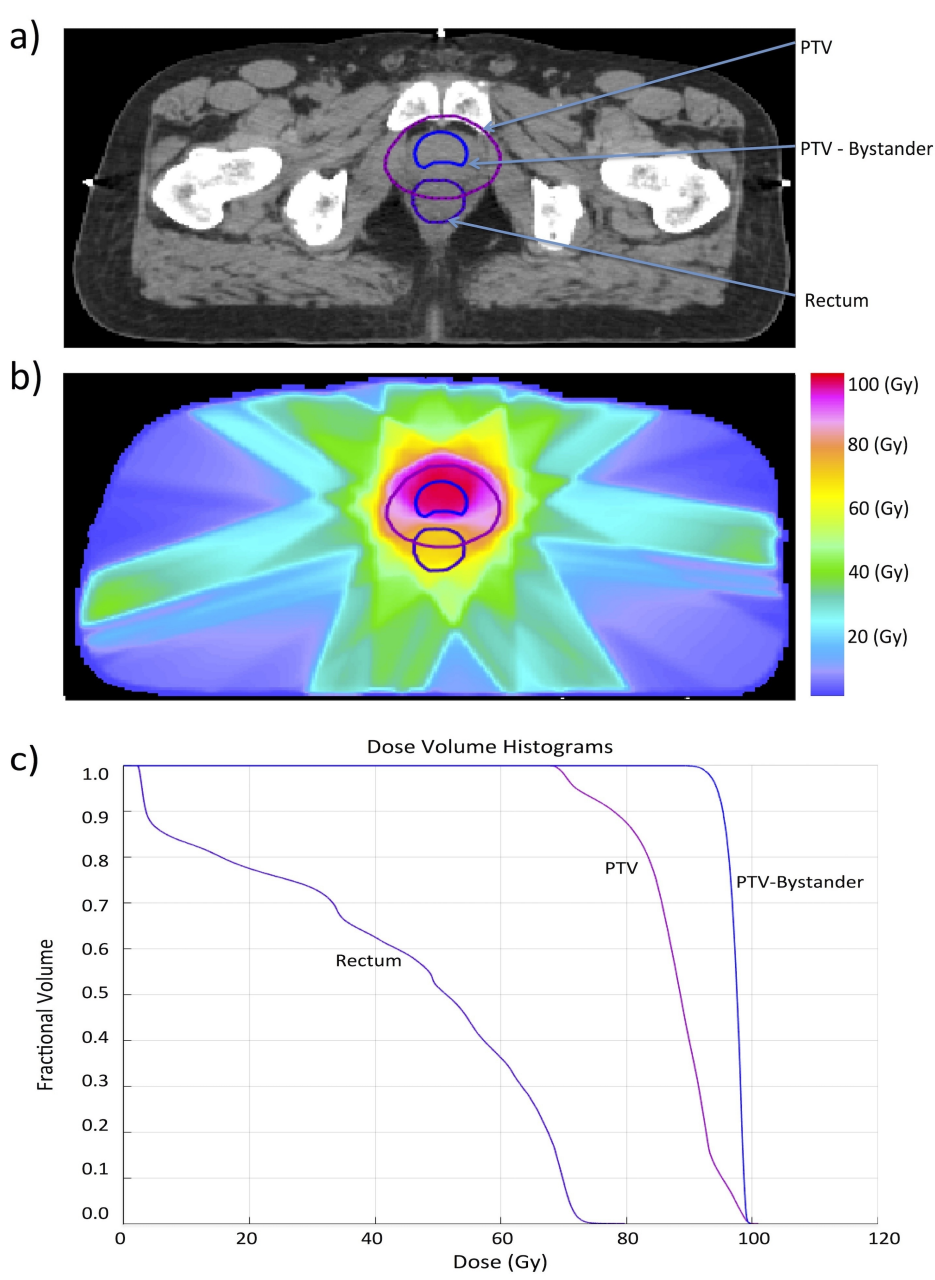
Biological optimization of a prostate cancer case a) Contouring of the planning target volume (PTV), rectum and a region in which bystander effects are to be included. b) Dose map for the biologically optimized treatment plan. c) Cumulative dose-volume histogram corresponding to the biologically optimized treatment plan.

Computed tomography (CT) images for a patient with prostate cancer were transfered to our research treatment planning system (Eclipse 13.7, Varian Medical Systems, Palo Alto, CA). The entire prostate was contoured and a planning target volume (PTV) was generated by margin expansion. The rectum was also contoured and included as an organ at risk in the biological optimization. In addition, a small intermediate/high-risk region within the PTV consisting of 1004 voxels was contoured in order to study the effects of bystander interactions (PTV-Bystander). A representative CT slice showing the contouring of these three structures is shown in Figure 1 (a). Treatment planning was performed using a planar, equiangular arrangement of seven X-ray beams. Dose optimization utilized a 3-dimensional cubic grid, with each voxel of dimension, $a^3 = .1270 \times .1270 \times .1270$  $cm^3$. The TCP for the PTV and PTV-Bystander structures and NTCP for the rectum were constructed by equating the input parameters of the linear-quadratic Poisson model as implemented in Eclipse, to the parameters of the logistic model in equation (14). The values of $D_{50}$ for the PTV and PTV-Bystander were taken from Levegrün [31] to be $64.5$ Gy (favorable risk) and $72.8$ Gy (intermediate/high risk) respectively. For the rectum, we chose a $D_{50} = 81.9$ Gy as is appropriate for late rectal bleeding as determined by Rancati, et al. [32]. For simplicity, we used a $\gamma_{50} = 5$ for all three structures, which is within the range of parameter values used in the literature [3]. In addition to $D_{50}$ and $\gamma_{50}$, the linear-quadratic Poisson model requires an estimate of the ${\alpha}/{\beta}$-ratio as input. We chose the values ${\alpha}/{\beta} = 10$ Gy for the prostate and ${\alpha}/{\beta} = 3$ Gy for the rectum. From the TCP and NTCP, the uncomplicated tumor control probability (UTCP) was constructed and optimized using the biological modeling package in our research treatment planning system. The prescription dose was chosen as to guarantee a $TCP =  1.0$  through the target region. The resulting dose matrix and the cumulative dose-volume histogram for the three contoured structures is shown in Figure 1.

From this dose matrix we have constructed the tumor probability distribution (equation (27)) within the region PTV-Bystander, for the different model parameters discussed below. Because the bystander interactions correlate each voxel with all other voxels, it is necessary to explicitly carry out the sum in the tumor partition function (equation (28)), over all $2^{1004}$ possible configurations. In our calculations, this was accomplished using Markov chain Monte-Carlo, with a Metropolis sampling algorithm [33]. Details of the simulation are provided in Appendix A.

From the tumor probability distribution, it is possible in principle to calculate the correlated N-voxel TCP. In practice, this is numerically intractable because the signal to noise ratio for the correlated N-voxel TCP is extremely small for a Monte Carlo sampled probability distribution. Instead, we have chosen to work with the average TCP per voxel, $TCP_{avg} = \frac{1}{N} \sum_{i=1}^N TCP_{i},$    (32) which is more easily numerically calculated and contains essentially the same information as the N-voxel TCP. $TCP_{avg}$ is analogous to the order parameter or average magnetization of the Ising model introduced in equation (11). Additionally, all our simulations use the linearized bystander interaction in equation (31) rather than the non-linear interaction of equation (30), which is numerically less stable.

As can be seen from Figure 1 (c) PTV-Bystander receives a nearly homogeneous dose of approximately 97 Gy since we have planned for a $TCP = 1.0$ through out this region. However, note that the rectum is still well spared. Since $TCP(D)=1$ within the entire PTV-Bystander structure, bystander interactions are not relevant at this dose level. In the following example, we will decrease the dose beginning from the dose level giving a $TCP = 1.0$ to determine the effect of bystander interactions on the corresponding decrease in TCP.

Bystander interactions between voxels in case of homogeneous dose distribution

We now study the effect of bystander interactions between voxels when a nearly homogeneous dose is delivered. By adding or subtracting a constant dose $\Delta D$ from each dose voxel of the dose matrix covering PTV-Bystander ​​​​​​​in Figure 1 (b), we can study the effect of varying the average dose to PTV-Bystander, while still maintaining a nearly homogeneous dose distribution within it. With the chosen biological parameters, one finds that without bystander effects, $TCP_{avg} \approx 1$ as long as the dose  is $\geq 87$ Gy, while $TCP_{avg}$ starts to decrease when the dose drops below this dose level. Therefore, we have chosen 87 Gy as the origin of $\Delta D$. One can interpret negative values of $\Delta D$ as providing information about how much of a decrease in dose can be tolerated, before the TCP falls appreciably below 1. Positive values of $\Delta D$ correspond to excess dose beyond that required to achieve complete sterilization. For the dose distribution shown in Figure 1, $\Delta D = 10$ Gy, which implies that we deliver an excess dose of 10 Gy beyond that required to achieve almost complete sterilization. To convert between absolute dose, $D_{abs},$ and $\Delta D$, one simply uses the relation $D_{abs} = 87$ Gy $+ \Delta D$.

**Figure 2:**
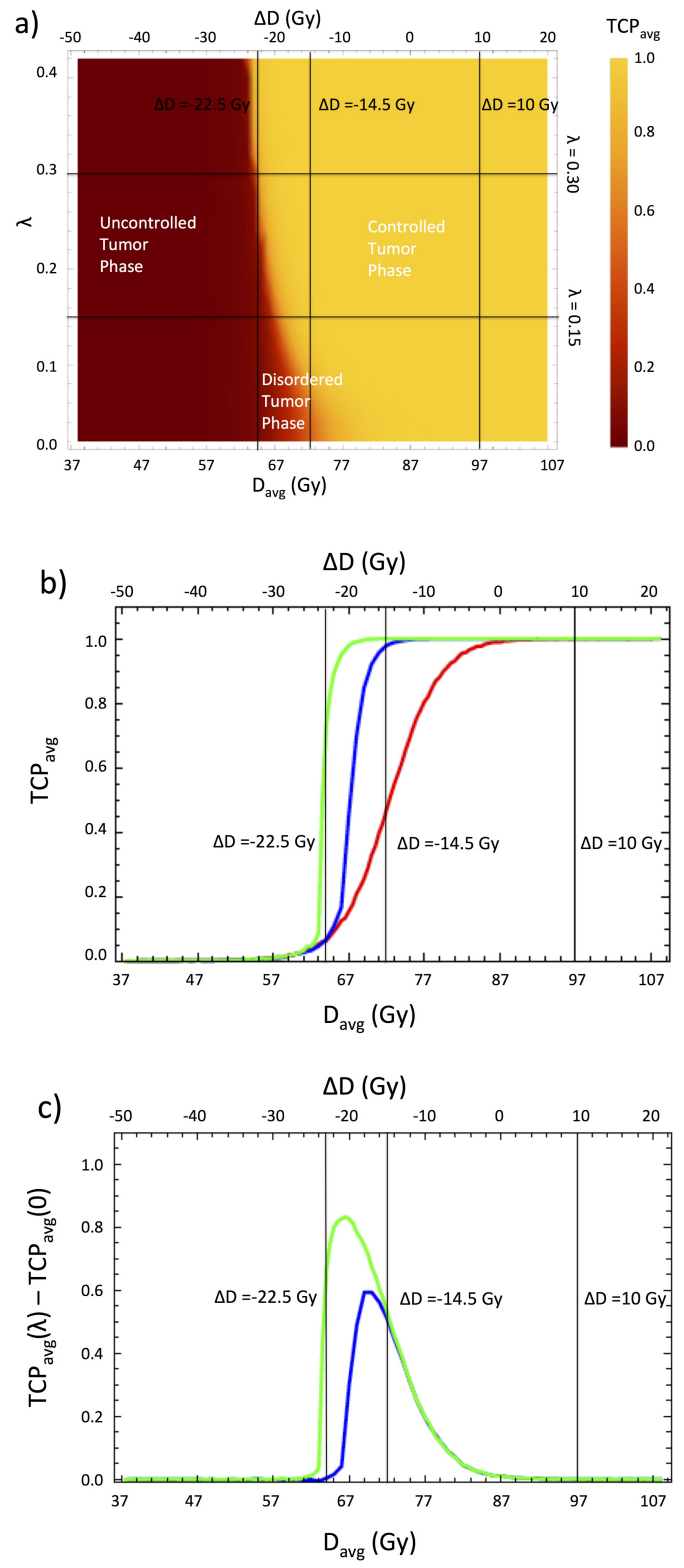
Dose response to a homogeneous dose distribution within PTV-Bystander a) Two dimensional color density plot of the average tumor control probability ($TCP_{avg}$) as a function of $\Delta D$ and $\lambda$. b) $TCP_{avg}$ plotted as a function of $\Delta D$ for: $\lambda = 0$ cm (red curve), $\lambda = 0.15$ cm (blue curve) and $\lambda = 0.30$cm (green curve). c) Difference of $TCP_{avg}$ for $\lambda = 0.15$ cm (blue curve) and $\lambda = 0.30$ cm (green curve) from $TCP_{avg}$without bystander effects ($\lambda = 0$).

To study the effect of different bystander interactions, we vary only the chemical diffusion length, $\lambda$, keeping the diffusion magnitude fixed at $J_0 =2$ Gy. This simplifies the analysis and in our simulations, we have found little qualitative difference between varying $\lambda$ and varying $J_0$. In Figure 2, the average TCP per voxel is plotted as a function of the dose difference $\Delta D$ from the optimized value, for different values of the chemical diffusion length. The case $\lambda = 0$ corresponds to non-interacting voxels. In our simulations, the case of $\lambda = 0.15$ cm corresponds to a diffusion distance of 1.2 voxels, implying that each voxel interacts strongly only with its immediate neighboring voxels. $\lambda = 0.30$ cm corresponds to a diffusion distance of $2.35$ voxels, so that each voxel interacts strongly with its nearest and next nearest neighbors.

As one can see from Figure 2 (b) that when bystander interactions are neglected, the dose response curve is sigmoidal and symmetric about the value $\Delta D = -14.5$ Gy, which corresponds to an absolute dose near the 50 percent lethal dose, $D_{50} = 72.8$ Gy. For the optimized dose, $\Delta D = +10$ Gy, all voxels are clearly sterilized, while for $\Delta D = -22.5$ Gy corresponding to an absolute dose of approximately 64.5 Gy, very few voxels are sterilized. One sees from Figure 2 (c) that bystander interactions have the effect of shifting  $D_{50}$ to the left towards lower values. The lowering of $D_{50}$ arises because the bystander interactions lead to an increased radio-sensitization of each voxel, as adjacent voxels are killed. Indeed, when $\Delta D = -14.5$ Gy only 50 percent of non-interacting voxels are sterilized, while nearly all voxels are sterilized when interactions are included (cf. Figure 2 (b) and Figure 2 (c)). In addition to radio-sensitization, bystander effects lead to an increase in the steepness of the dose response curve. This arises because the bystander interactions cause the tumor to be sterilized as a collective unit, rather than a parallel structure composed of independent clonogens. At low dose, essentially all voxels are unsterilized and bystander effects are negligible. However, as the dose is increased, a “critical mass” of voxels are sterilized and bystander effects become sufficiently strong to sterilize the vast majority of remaining voxels with little additional dose. Voxels near the edge of the tumor will incur fewer bystander interactions than those in the bulk and account for the additional dose required to achieve 100 percent sterilization. This surface effect, which becomes less important for large tumors, accounts for the asymmetry in the dose response curves about $D_{50}$ seen in Figure 2 (b).

**Figure 3:**
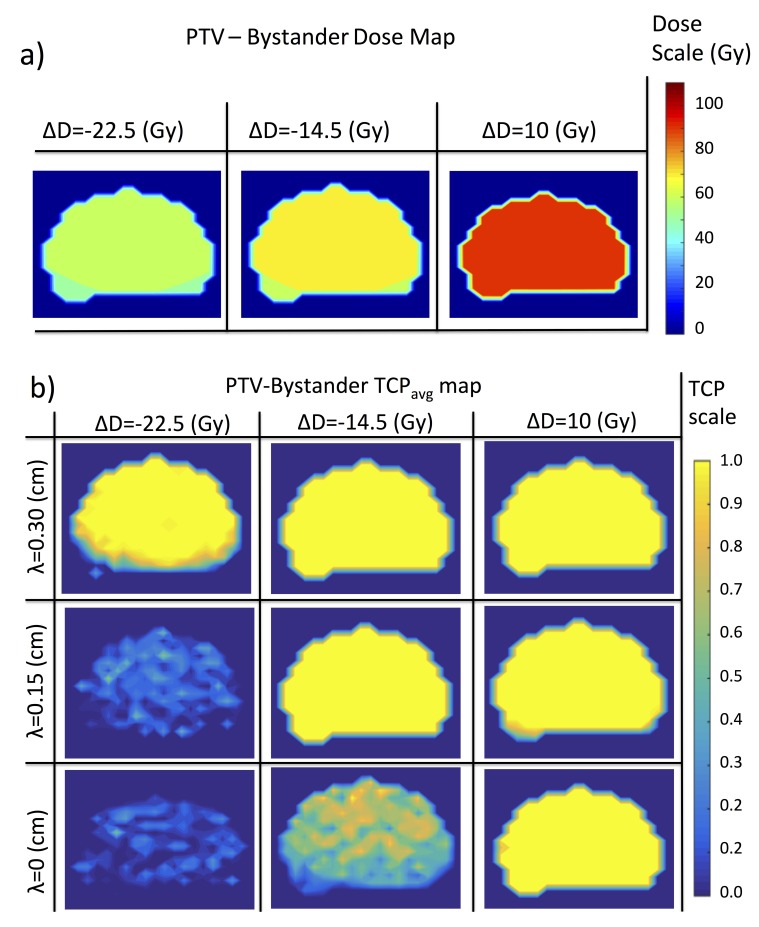
Dose and tumor control probability (TCP) maps for a homogeneous dose distribution a) Dose distribution within PTV-Bystander for the selected axial CT slice shown in Figure 1. The homogeneous shifts in dose, $\Delta D$, correspond to the vertical lines in Figure 2. b) TCP maps are plotted for the points of intersection of the vertical lines (constant $\Delta D$) and horizontal lines (constant $\lambda$) in the phase diagram of Figure 2(a).

The behavior seen in the dose response curves of Figure 2 (b), in which there is a collective change in the ordering of a system as interactions and external parameters are varied, is reminiscent of a phase transition from statistical mechanics. In our case, the interactions are bystander interactions, the varied external parameter is the dose and the average TCP per voxel, $TCP_{avg}$, plays the role of an order parameter. This analogy is made particularly apparent by looking at the tumor phase diagram in Figure 2 (a). The tumor consists of three different phases: 1) an ordered, “uncontrolled” phase, in which all voxels are unsterilized and hence $TCP_{avg}=0$, 2) a disordered phase, in which voxels are partially sterilized and therefore $0 &lt; TCP_{avg} &lt; 1$, 3) an ordered or “controlled” phase, in which all voxels are sterilized, with $TCP_{avg}=1$. In the phase diagram we see that as the range of bystander interactions is increased, the disordered phase begins to vanish and there is a nearly discontinuous transition between the sterilized and unsterilized phases. As the tumor temperature is held fixed and the interactions are increased, one sees an order-disorder transition as the ratio of the interaction strength to the temperature increases. In Figure 3, the dose distribution and the single-voxel TCP are plotted within the region PTV-Bystander for the selected axial slice shown in Figure 1. 

Note that when the tumor is already well controlled then bystander interactions have little to no effect (cf. right most panel Figures 3 (a) and Figure 3 (b)). However, near $D_{50}$ where the dose response curve is changing rapidly, even weak bystander interactions can have a large effect (cf. middle pannel of Figure 3 (b)). At doses much lower than $D_{50}$, weak bystander interactions are no longer relevant because the tumor is well within the uncontrolled phase. In Appendix B, we show that for large tumors, bystander interactions can be neglected provided the condition,

$|D - D_{50}| \gg 8 \pi J_0 \left[ \frac{\lambda}{a} \right]^3,$    (33) is satisfied. This provides a simple means to determine whether or not it is necessary to include voxel-voxel interactions in a given biological-based IMRT calculation, when the dose distribution is nearly homogeneous. The right hand side of equation (33) is proportional to $J_0$, which quantifies the amount of radio-sensitization that a sterilized voxel imparts to other voxels and  $\lambda^3$, which is the chemical diffusion volume. It is inversely proportional to the voxel volume $a^3$. The ratio $\left[ \frac{\lambda}{a} \right]^3$ is therefore equal to the number of voxels that are radio-sensitized by bystander interactions in the vicinity of a single sterilized voxel. As expected, when this quantity is large, a well optimized dose distribution (large $|D-D_{50}|$) is required in order for bystander effects to be neglected. Equation (33) also predicts that for a given chemical diffusion length, as the voxel size is increased bystander interactions become less important. This is because in the limit $a \gg \lambda$, any bystander interactions are already included in the single-voxel biological parameters $D_{50}$ and $\gamma_{50}$. Thus, in this limit it is possible to include bystander interactions in single voxel parameters, rather than as explicit voxel-voxel interactions.

## Discussion

In the present study, we have established a connection between the mathematical formulation of biological-based IMRT and the Ising model of statistical mechanics. Although we have focused on bystander effects in TCP modeling here, the general link between biological-based IMRT and statistical mechanics opens several future research directions.

**Figure 4:**
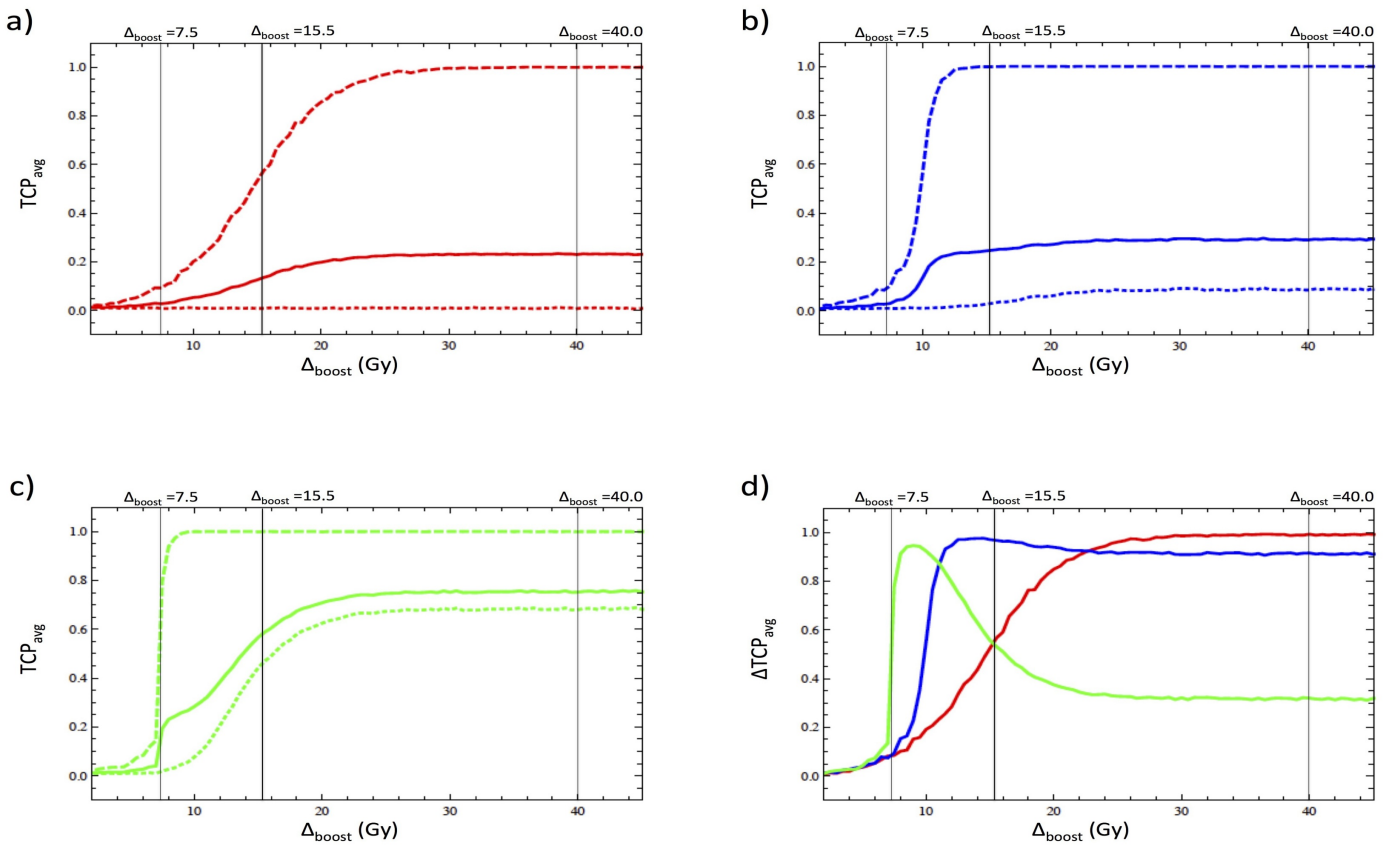
Dose response to an inhomogeneous dose distribution a) Average tumor control probability ($TCP_{avg}$) within the boosted region (dashed), the unboosted region (dotted) and the entire PTV-Bystander structure (solid) as a function of the boosting dose, for $\lambda = 0$. b) The same as graph (a), but with $\lambda = 0.15$ cm. c). The same as graph (a) and graph (b), but with $\lambda = 0.30$ cm. d) The difference in $TCP_{avg}$ between the boosted and unboosted regions for $\lambda = 0$ (red), $\lambda = 0.15$ cm (blue) and $\lambda = 0.30$ cm (green).

For completeness we now consider the effect of bystander interactions for the case of an inhomogeneous dose distribution in which a portion of PTV_Bystander is treated to a high dose level while a significant portion of PTV_Bystander is underdosed receiving a dose of 57 Gy (cf. Figure 4). For this purpose we first consider a fixed boost volume of 25 percent of PTV_Bystander and vary the boost dose from 0 to 45 Gy within it, which corresponds to varying the average dose in the *boost region* from 57 to 102 Gy.

As expected, when bystander interactions are neglected, we see from Figure 4 (a) that the average TCP in the *unboosted region* is completely unaffected by changes in the boost dose, while the average TCP for the entire PTV saturates at .25 for large values of the boost dose, as all voxels in the *boosted region* are sterilized, while those voxels in the *unboosted region* are not (cf. Ref. [6]). In Figure 4 (b) and Figure 4 (c) we see that bystander interactions cause the dose response curves in the *boosted region* to become steeper and shifted to the left relative to the dose response curve for the *boosted region* when neglecting bystander effects. This effect is due to interactions between voxels within the *boosted region* and is expected based upon the discussion of the effect of bystander interactions on homogeneous distributions discussed above. In addition to changing the dose response in the *boosted region*, we see that bystander interactions cause the average TCP in the *unboosted region* to increase as well. This effect is fairly small for $\lambda = 0.15$ cm, but becomes quite appreciable for $\lambda = 0.30$ cm. This effect arises because when bystander interactions are included, as the boost dose is increased, voxels near the boundary of the *boosted region* begin to sterilize nearby voxels in the unboosted region through bystander interactions. Biologically, this effect may be due to diffusion of chemical factors from the *boosted* region to the *unboosted *region, leading to an increase in indirect cell killing [20, 23], and has therefore been termed “biological penumbra” [21]. 

To better understand the biological penumbra, we have plotted in Figure 4 (d) the $\Delta TCP$, which is the difference in average TCP between the *boosted* and *unboosted* region. We see that with bystander interactions included, there is an initial rapid increase in the $\Delta TCP$ to a maximum value as voxels in the boosted region are sterilized with little effect on the unboosted region. As the *boost* dose is further increased, the average TCP in the *boosted* region saturates, but the biological penumbra continues to increase in size, which leads to a decrease in the $\Delta TCP$. Finally, as the boost dose is further increased, the biological penumbra reaches a maximum size and the $\Delta TCP$ asymptotes to a constant value. 

Mechanistically, the formation of the biological penumbra can be understood from the behavior of the voxels on the boundary of the *boost region*. Voxels on the boundary experience less radio-sensitization from bystander interactions than those in the bulk, because they have fewer strongly irradiated voxels in their immediate vicinity. Therefore, although the average TCP in the *boost region* is nearly saturated for a small boost dose, a large additional dose is required to completely sterilize the voxels on its boundary. It is precisely these voxels on the boundary of the *boost* *region* that are responsible for generating the biological penumbra by interacting with voxels in the *unboosted region*. Once all voxels on the boundary of the *boost* *region* are sterilized, additional boost dose will no longer lead to an increase in the size of the penumbra.

**Figure 5:**
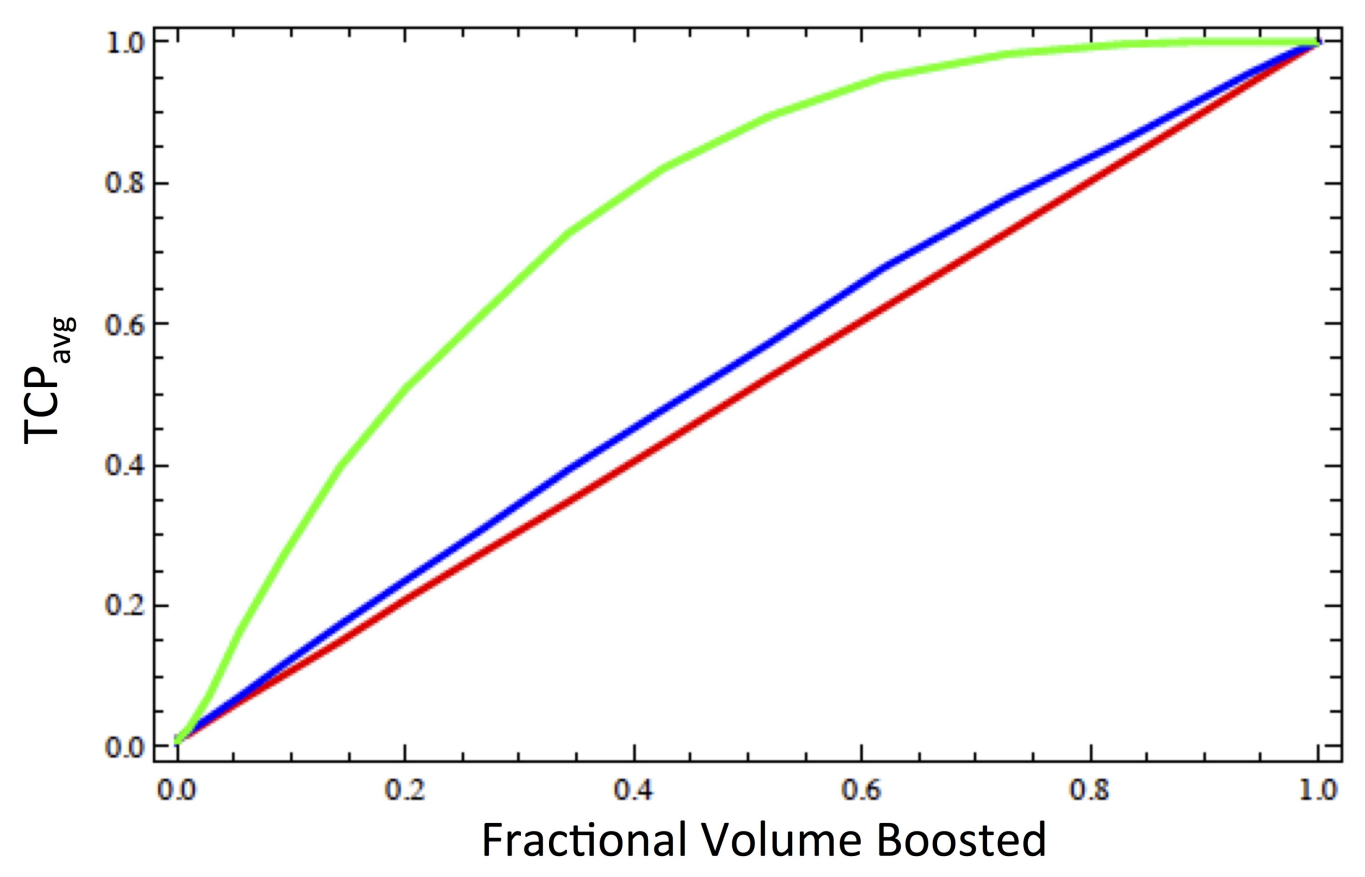
Dependence of the tumor control probability (TCP) on volume of the boosted region The average tumor control probability (${TCP}_{avg}$) within the entire structure PTV-Bystander is plotted as a function of the fractional volume of the region receiving a boosted dose ($\Delta_{boost} = 40$ Gy) for $\lambda = 0$ (red), $\lambda = 0.15$ cm (blue) and $\lambda = 0.30$ cm (green).

Up to this point we have held the boost volume fixed at 25 percent of the target volume, while varying the boost dose. In Figure 5, we hold the boost dose fixed at 40 Gy and instead vary the fractional volume of PTV-Bystander receiving the boost dose.

As expected without bystander interactions, the average TCP is simply equal to the fractional boost volume, as only those voxels in the boosted region are sterilized (cf. Ref [6]). However, when strong voxel-voxel interactions are included, it is possible to sterilize the entire PTV with less than 100 percent coverage, since as noted above a $\lambda = 0.15$ cm corresponds to a diffusion distance of 1.2 voxels, implying that each voxel interacts strongly only with its immediate neighboring voxels, while a $\lambda = 0.30$ cm corresponds to a diffusion distance of $2.35$ voxels, so that each voxel interacts strongly with its nearest and next nearest neighbors leading to this observed effect. Indeed, for a diffusion length of $\lambda = 0.30$ cm nearly 100 percent sterilization is achieved when only 80 percent of the PTV-Bystander receives the boost dose. This is because the remaining 20 percent of PTV-Bystander are well within the biological penumbra and therefore sterilized through indirect cell killing.

Bystander interactions represent one example where voxel-voxel interactions can be used to go beyond the assumption of a strictly parallel tumor architecture. For instance, the inclusion of a relative seriality parameter can be regarded as an interaction between voxels [34]. Other examples that can be modeled as voxel-voxel interactions include volume effects [35], in which the TCP is dependent upon the absolute volume of irradiated tissue as well as correlations between tumor and normal tissues, previously modeled using a non-zero delta parameter in the UTCP [36]. In addition to the TCP, it is possible to map the parameters of a logistic model for the NTCP to an Ising-like model in an analogous manner, and the maximization of the UTCP, which is equivalent to minimization of $-\ln\left( UTCP \right)$, can be formulated as the minimization of a free energy function analogous to equation (4), subject to constraints on the external beam geometry.

## Conclusions

Statistical mechanics has a rich history, with many established techniques and computational methods for solving the problem of optimizing the free energy of an interacting system subject to certain constraints. This opens the possibility that techniques from statistical mechanics can be applied to biological-based IMRT and/or used to guide development of optimization algorithms, especially when voxel-voxel interactions are relevant.

## Appendices

 A. Monte Carlo simulation

The biologically-optimized dose distribution was exported and used to generate the dose-matrix within the region PTV-Bystander. The dose-matrix is an indexed array with 1004 entries of the form $\{ D_{i,j,k} \}$, which specifies the value of the dose at each voxel. Here, $i$, $j$ and $k$ are positive integers specifying the coordinates of the voxel on a cartesian grid. The cartesian coordinates are related to the integers according to $x = a\times i$, $y= a\times j$ and $z = a \times k$, where $a = 0.1270$ cm. As input to the model, we choose to convert the dose matrix to a fractional effective dose-matrix $\{ FED_{i,j,k} \}$, which accounts for differences from the fractionation schedule used to derive the biological parameters [37]. This is done using the formula: 

$FED_{i,j,k} = n D_{i,j,k} \frac{\left[ \frac{\alpha}{\beta} + D_{i,j,k}  \right]}{\left[\frac{\alpha}{\beta} + f_s \right]}, \label{effec}$    (A.1)

where $n$ is the number of fractions and $f_s$ is the reference fraction size. In the present example $n = 35$, $f_s = 2$ Gy and $\frac{\alpha}{\beta} = 10$ Gy. From $\{ FED_{i,j,k} \}$ one can compute the interaction-matrix, which is an indexed array of $1004^2$ entries of the form $\{ J_{i,j,k; q,r,s} \}$ containing the bystander interactions between every pair of voxels. The explicit elements of the interaction matrix are obtained according to equation (31) as:

$J_{i,j,k; q,r,s} = J_0 \left[    \frac{1}{1 + e^{-\left[ \frac{4 \gamma_{50}}{D_{50}}\right] (FED_{i,j,k} - D_{50}) }}   \right]e^{-\frac{a}{\lambda} \sqrt{(i-q)^2 + (j-r)^2 + (k-s)^2}}. \label{int_mat}$    (A.2)

The goal of the Monte Carlo simulation is to iteratively sample the probability distribution in equation (27). We denote the configuration of the voxels after t iterations of the algorithm as an array of the form $\{ \sigma_{i,j,k}^t \}$, which is a string of length 1004 and consists of 0's and 1's. The string $\{ \sigma_{i,j,k}^{t+1} \}$ is generated from the string $\{ \sigma_{i,j,k}^{t} \}$ using the Metropolis algorithm as follows: 

1) Construct the tumor energy of the configuration $\{ \sigma_{i,j,k}^{t} \}$:

$E^t = -\sum_{i,j,k} \sum_{q,r,s} \sigma_{i,j,k}^{t} J_{i,j,k; q,r,s} \sigma_{q,r,s}^{t} + \sum_{i,j,k} (D_{50} - FED_{i,j,k}) \sigma_{i,j,k}^{t}.$    (A.3)

2) Choose a single point $(x = a\times i, y= a\times j, z = a \times k)$ on the grid at random.

3) Propose the following trial: If the voxel at this point has configuration $\sigma_{i,j,k}^{t} = 0$ then set $\sigma_{i,j,k}^{t+1} = 1$. If on the other hand $\sigma_{i,j,k}^{t} = 1$, set $\sigma_{i,j,k}^{t+1} = 0$.

4) Compute the change in energy $\Delta E = E^{t+1} - E^{t}$ due to the change in step 3).

5) If $\Delta E &lt; 0$, accept the trial.

6) If $\Delta E > 0$, accept the trial with probability $e^{-\left[ \frac{4 \gamma_{50}}{D_{50}} \right] \Delta E}$.

7) If the trial is accepted, the change proposed in step 3 becomes the new configuration. If the trial is rejected, simply set $\sigma_{i,j,k}^{t+1} = \sigma_{i,j,k}^{t}$.

It can be shown that if the above algorithm is iterated to convergence, the configurations $\{ \sigma_{i,j,k}^{t} \}$ at subsequent iterations are distributed according to the probability distribution in equation (27). In our simulations we choose the initial configuration, $\sigma_{i,j,k}^0$, to be a random string. Starting from this configuration, we apply the metropolis algorithm for 14,000 iterations, which is sufficient to reach convergence. The algorithm is then repeated for another 6000 iterations to sample the probability distribution. The configurations obtainied for $14000 \leq t \leq 20000$ can then be used to approximate the tumor partition function according to:

$Z = \sum_{\{ \sigma \}} e^{-\left[ \frac{4 \gamma_{50}}{D_{50}} \right] E(\{ \sigma \})} \approx  \frac{1}{6000} \sum_{t=14,000}^{20,000} e^{-\left[ \frac{4 \gamma_{50}}{D_{50}} \right] E^t}.$    (A.4)

In equation (A4) the sum over tumor configurations has been numerically approximated by a sum over the sampled iterations. From derivatives of this partition function, the single-voxel TCP and the average TCP can be numerically calculated. Equivalently, one can calculate these quantities directly from the sampled probability distribution as follows:

$TCP_{ijk} = \sum_{\{ \sigma \}} P(\{ \sigma \})\sigma_{i,j,k} \approx \frac{1}{6000} \sum_{t=14,000}^{20,000} \sigma_{i,j,k}^{t}$    (A.5)

and $TCP_{avg} = \frac{1}{1004} \sum_{i,j,k} TCP_{ijk}$. The latter procedure was used to generate the data in our simulations.

B. Derivation of equation (33)

Assuming a homogeneous dose distribution, we can write equation (28) for the tumor partition function as:

$Z = \sum_{\{ \sigma \}}  e^{-\left[ \frac{4 \gamma_{50}}{D_{50}} \right] \left[ - \sum_{{i,j}}^{N} J_{ij} \sigma_i \sigma_j  - (D - D_{50} )  \sum_{i=1}^{N}  \sigma_i \right]} = \sum_{\{ \sigma \}} e^{\left[ \frac{4 \gamma_{50}}{D_{50}} \right] (D - D_{50} )\sum_{i=1}^{N}  \sigma_i  \left[1 +   \sum_{{j}}^{N} \frac{J_{ij}}{(D - D_{50} )}\sigma_j  \right]}.\label{tumor_partition_S1}$    (B.1)

For bystander interactions to be negligible the approximation,

\(Z \approx  \sum_{\{ \sigma \}} e^{\left[ \frac{4 \gamma_{50}}{D_{50}} \right] (D - D_{50} )\sum_{i=1}^{N}  \sigma_i }, \label{tumor_partition_S2}\)    (B.2)

must hold, i.e. it must be possible to approximate the tumor partition function with the non-interacting partition function. From a Taylor expansion of equation (B.1), we see that this is valid provided,

$1 \gg \sum_{{j}}^{N} \frac{J_{ij}}{|D - D_{50} |}\sigma_j ,$    (B.3)

or equivalently by using equation (30), $|D - D_{50} | \gg \sum_{{j}}^{N} J_0 TCP_{i}  e^{-\frac{|\vec{r}_i - \vec{r}_j|}{\lambda}} \sigma_j.$    (B.4)

Since $TCP_{i}$ and $\sigma_j$ are bounded between 0 and 1, equation (B.4) is clearly satisfied provided the stricter inequality,

$|D - D_{50} | \gg \sum_{{j}}^{N} J_0   e^{-\frac{|\vec{r}_i - \vec{r}_j|}{\lambda}} ,$    (B.5)

holds. If one assumes that the tumor volume $V_T$ greatly exceeds the voxel dimension $a$, one can replace the discreet sum by an integral according to,

$\sum_{{j}}^{N} J_0   e^{-\frac{|\vec{r}_i - \vec{r}_j|}{\lambda}} \approx \frac{J_0}{a^3} \int_{V_T} d^3r_j e^{-\frac{|\vec{r}_i - \vec{r}_j|}{\lambda}}$     (B.6)

In general, this integral will depend on the specific geometry of the tumor. However, if the dimensions of the tumor are much larger than $\lambda$ one can approximate the integral in eq. (B.5) by an integral over all space. In spherical coordinates this becomes:

\( \int_{V_T} d^3r_j e^{-\frac{|{\vec{r}}_i - {\vec{r}}_j|}{\lambda}} \approx 4 \pi \int_{0}^{\infty} dr (r^2) e^{-\frac{r}{\lambda}} = 8 \pi \lambda^3 \)    (B.7)

Substituting this result into equation (B.6) and then substituting equation (B.6) in equation (B.5) yields equation (33).
